# Comment on: Effects of COVID-19 on human placentas in the second and third trimester

**DOI:** 10.61622/rbgo/2024rbgo88

**Published:** 2024-12-04

**Authors:** Nayara Ribeiro Máximo de Almeida, Mateus Augusto Felix de Melo, Pâmela Marillac Rodrigues Feijó de Melo, Julio Martinez Santos, Johnnatas Mikael Lopes

**Affiliations:** 1 Universidade Federal do Vale do São Francisco Paulo Afonso Bahia Brazil Universidade Federal do Vale do São Francisco, Paulo Afonso, Bahia, Brazil.

Recent evidence demonstrates na increase in negative maternal and neonatal outcomes in cases of SARS-CoV-2 infection, such as greater severity of the disease, need for mechanical ventilation and longer hospitalization in intensive care units.^(1,2)^ The greater severity of infectious diseases in pregnancy occurs due to anatomical and immunological changes, such as a change in the CD4 cell phenotype from the TH2 subgroup to TH1, a decrease in the function of natural killer cells and a reduction in plasma cell dendritic cells, with the first and third trimesters of pregnancy being the periods of greatest inflammatory activity and susceptibility to diseases.^(3)^ Furthermore, there are obstetric and neonatal impacts associated with COVID-19 infection during pregnancy, such as premature rupture of ovular membranosa, prematurity, fetal distress, increased number of cesarean sections, tachypnea, neonatal sepsis and death maternal.^(4,5)^

COVID-19 infection can also cause damage to the placenta, especially if it comes from the delta variant. It is known that COVID requires ACE II and TMPRSS2 receptors to initiate and facilitate infection, but these receptors are poorly expressed in placental cells, which reduces the structural damage caused by the disease.^(6)^ However, this factor does not prevent disease. Placental, since signs of maternal-fetal vascular malperfusion, zones of avascular villi, villous thrombi and infarction and villous edema can be observed.^(4)^ In addition to histiocytic intervillositis, deposition of perivillous or subchorionic fibrin, trophoblastic necrosis and increased incidence chorangiosis.^(5)^ These changes are mainly due to cellular reactions caused by the death of the virus, the action of anti-SARS-CoV antibodies, the local inflammatory process and syncytiotrophoblastic ischemia, without necessarily having a placental infection.^(3)^

The study by Helmi and Al-Badri,^(7)^ entitled "Association of placental histopathological findings with COVID-19 and its predictive factors" points out such structural changes in the human placenta. However, despite the great relevance of the study and its findings, methodological errors were identified when constructing the analysis of possible risk factors for the structural changes highlighted.^(8)^

Furthermore, Helmi and Al-badri presented a statistical model to explain the prediction of histopathological findings in placentas, making it possible to highlight two mistakes. The first of these is the lack of presentation of the effects of variables that were shown to be related to the outcome in the bivariate analysis, such as "Timing of infection during pregnancy (Weeks)", "Random blood glucose (mg/dl)", "Lymphocytes (%)", making the model of low credibility. The second misconception is the application of binary logistic regression modeling to longitudinal retrospective cohort designs. This statistical technique creates a measure of effect, odds ratios, oversized, in a punctual and interval manner, as can be seen in the variable "COVID-19 diagnosis to delivery (Weeks)", which presents a wide confidence interval (95% CI: 1.19 – 6.36) and, presumably, the odds, at a value of 2.75, were overestimated, given the possibility of being influenced by other variables not included in the model.

Therefore, we suggest that the data presented from predictive modeling be estimated using Poisson Robusta or Cox regressions, which produce unbiased effect measures in prospective and retrospective cohort studies.
